# The condition of hair follicles produced by different punching methods during FUE surgery

**DOI:** 10.1111/jocd.16537

**Published:** 2024-08-16

**Authors:** Jino Kim, Yong‐Uk Ko, Kyu‐Ho Yi

**Affiliations:** ^1^ New Hair Plastic Surgery Clinic Seoul South Korea; ^2^ Division in Anatomy and Developmental Biology, Department of Oral Biology Human Identification Research Institute, BK21 FOUR Project, Yonsei University College of Dentistry Seoul South Korea; ^3^ Maylin Clinic (Apgujeong) Seoul South Korea

**Keywords:** follicular unit extraction, graft survival, hair transplantation, scalp inflammation

## Abstract

**Background:**

Achieving successful outcomes in hair transplant surgery involves various critical factors, including donor area harvesting, graft survival, and minimizing post‐operative complications. This study investigates the differences in grafts obtained using the rotary and oscillatory punch methods during follicular unit extraction (FUE) surgery.

**Methods:**

The study involved 15 patients undergoing FUE. Four 4 × 6 cm^2^ areas in two rows were selected for each patient, with each row utilizing a different punch method (rotary or oscillatory). The grafts were extracted and examined under a microscope, classified into single, double single, double, and triple categories. The total yield rate and average number of hairs per graft were measured and compared.

**Results:**

The average number of hair follicles per graft was 2.029 for the rotary method and 2.084 for the oscillatory method, indicating no statistically significant difference. However, the total yield rate was 88.3% for the rotary group and 90.5% for the oscillatory group, with the difference being statistically significant. In selected cases with soft scalps or deeper punch requirements, the oscillatory method showed significantly better results, with an average of 2.078 hairs per graft compared to 1.836 for the rotary method. The total yield rate in these cases was 91% for oscillatory and 86% for rotary.

**Conclusion:**

While the overall differences between rotary and oscillatory punches are minimal, the oscillatory punch is significantly more effective in cases with soft scalps or deeper punch requirements. Adhering to a structured guideline before extraction can help reduce the transection rate and increase the number of hairs per graft.

## 1 INTRODUCTION

Achieving successful outcomes in hair transplant surgery involves several critical factors. These include donor area harvesting, graft survival, and post‐operative complications, among others. This study aims to investigate the differences in grafts obtained using the rotary and oscillatory punch methods.^1^

**FIGURE 1:**
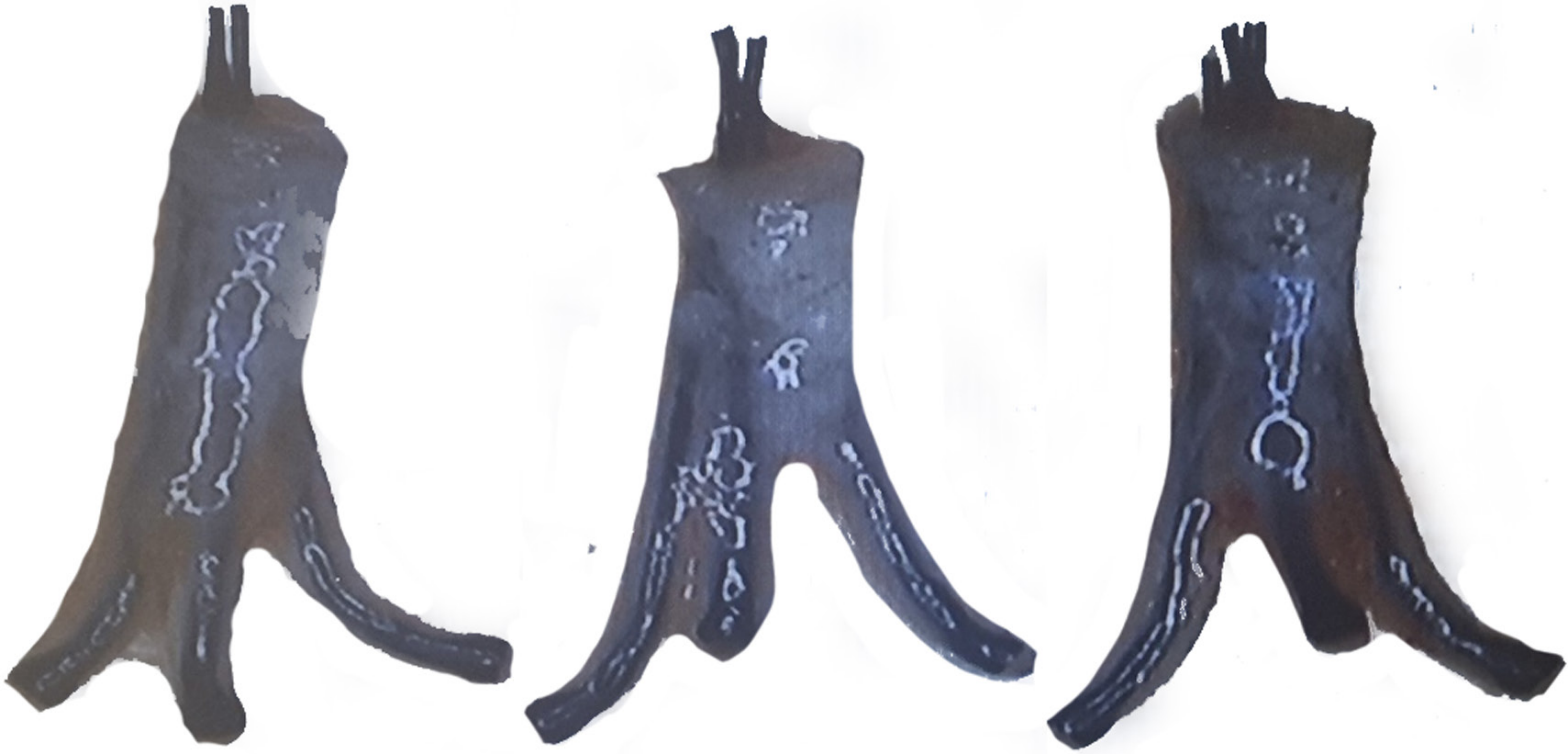
Splay describes the conical shape of hair growth, where the width of the hair increases toward the base.

To harvest optimal hair follicles, it is essential to understand their anatomical elements. Splay describes the conical shape of hair growth, where the width of the hair increases toward the base (Figure 1).^1^, ^2^

**FIGURE 2:**
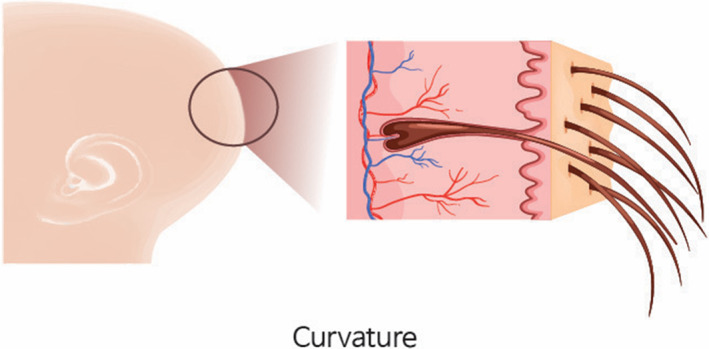
Curvature refers to the bending direction of the hair, with curlier hair exhibiting greater curvature.

**FIGURE 3:**
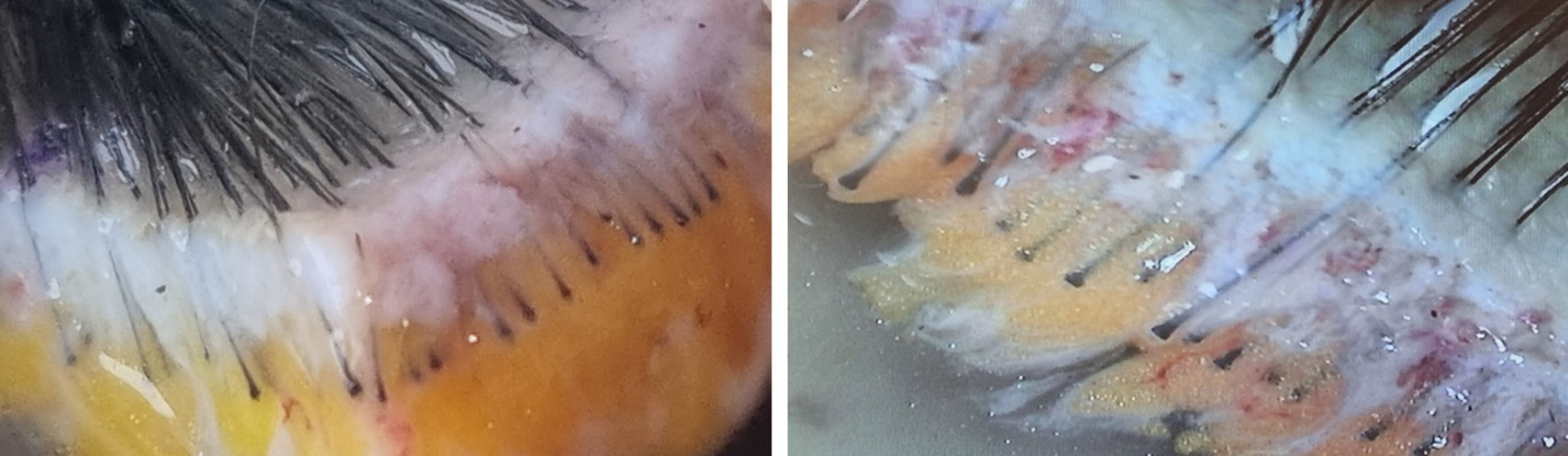
Occipital nerve block before the hair harvesting.

Curvature refers to the bending direction of the hair, with curlier hair exhibiting greater curvature (Figure 2). Tethering is considered one of the primary factors leading to varied harvesting results in non‐incisional hair transplants (Figure 3).

Tethering occurs due to several connections such as the dermal sebaceous glands, connective tissue surrounding the subcutaneous dermis of the follicle, and the arrector pili muscle. These connections are hypothesized to contribute to the tethering effect observed during follicle extraction.^3^, ^4^, ^5^

The rotary method involves continuously rotating the punch in one direction to extract the graft. To reduce the transection rate, the punch can be inserted to a minimal depth and its diameter can be increased. The depth of insertion and the diameter of the punch may vary depending on the individual case of each patient. The oscillatory method involves repeatedly changing the direction of the punch rotation.^6^ The primary theoretical advantage of the oscillatory method is that the small arc of rotation generates less torsional force, thereby avoiding complete twisting of the follicle. This approach is believed to reduce damage to splayed follicles and those with significant curvature, allowing for deeper penetration into the skin.^7^

The goal of this study is to investigate the differences in the condition of hair follicles produced by the rotary and oscillatory punching methods during follicular unit extraction (FUE) surgery. Achieving successful outcomes in hair transplant surgery involves several critical factors, including donor area harvesting, graft survival, and post‐operative complications, among others.

## 2 MATERIALS AND METHODS

**FIGURE 4:**
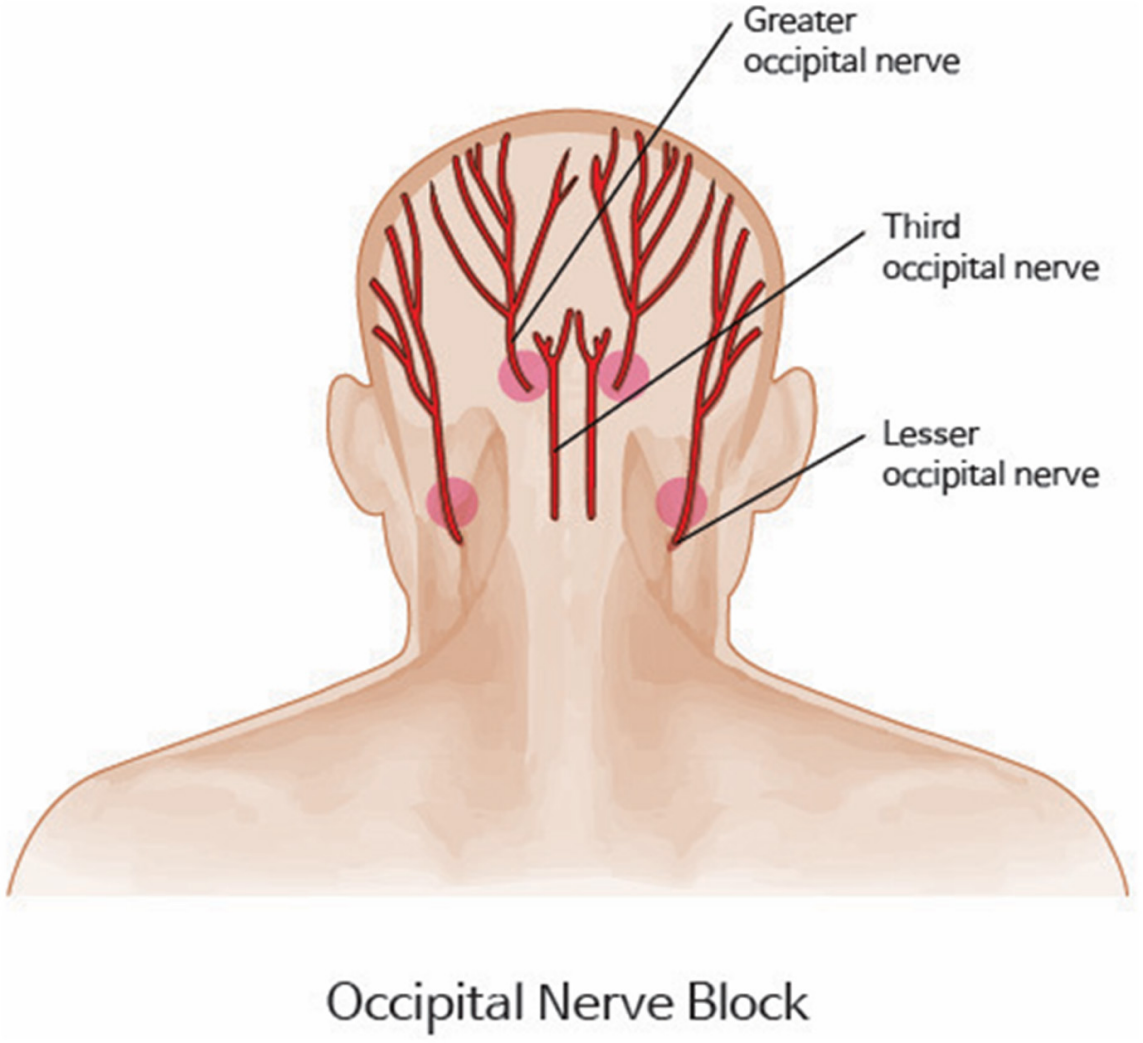
Idiopathic occipital fibrosis (IOF) refers to a condition where there is a subtle, chronic inflammation in the occipital area from which hair is harvested. This inflammation can cause tethering, making it difficult to extract hair.

**FIGURE 5:**
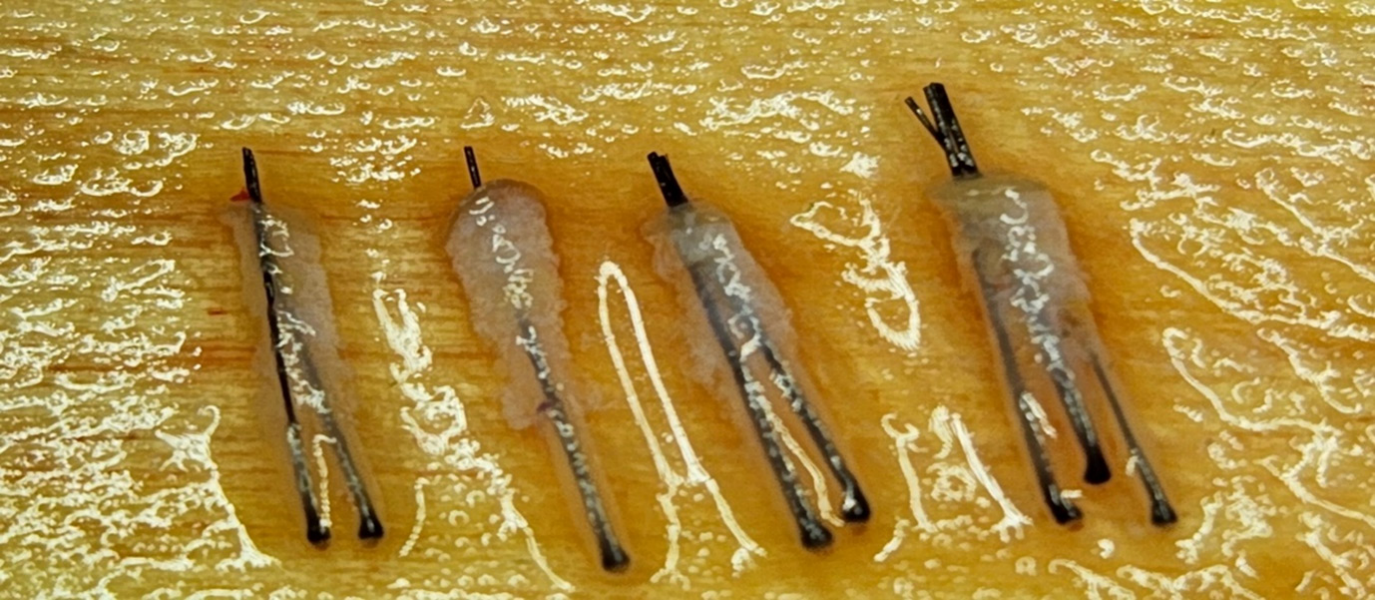
From the left the harvesting was done in double single, single, double, and triple.

This study was conducted in accordance with the Declaration of Helsinki. In this study, 15 patients who underwent FUE were selected. For each patient, four areas of 4 × 6 cm^2^ were chosen in two rows, with each row utilizing a different punch method (either rotary or oscillatory). The occipital nerve block is conducted before harvesting (Figure 4). To minimize variables, the rows were classified in a zigzag pattern for harvesting. The grafts were extracted while the patient was in a supine position, with the surgeon seated at the head of the patient, beginning extraction from the right side to the left. Specifics about the diameter (1.0 mm), depth (3–4 mm), and characteristics of the punches (round sharp punch) used in both the rotary and oscillatory methods. The harvested follicles from each area were examined under a microscope and classified into single, double single, double, and triple categories (Figure 5). Additionally, the total yield rate and the average number of hairs per graft were measured and compared. The statistical test conducted for comparing the differences between the rotary and oscillatory methods was a paired sample *t*‐test.

The average follicle length and punch depth were recorded for each case, along with the specific punch method used (rotary or oscillatory). The rotary method was performed at a consistent speed of 10 000 rpm across all cases. The oscillatory method was characterized by a 270‐degree rotation and a 7 rms setting in most cases, except for cases 11–15, where an 11 rms setting was used. The cases included a mix of conditions: “soft tissue” cases, requiring a more delicate approach, and “deep punch” cases, indicating a need for greater depth penetration (from depth of 4–6 mm). The average follicle length, punch depth, punch technique (soft/deep), rotary speed (rpm), and oscillatory parameters (degree and RMS) for cases are summarized in Table 1.

**TABLE 1:** The table shows the average follicle length, punch depth, punch technique (soft/deep), rotary speed (rpm), and oscillatory parameters (degree and RMS) for various cases.

	Average follicle length (mm)	Punch depth (mm)	Soft/Deep punch	Rotary	Oscillatory
Case 1	6.15	4.05		10 000 RPM	180°, 7 rms
Case 2	6.05	4.5	Deep punch	10 000 RPM	270°, 7 rms
Case 3	6.5	4.3		10 000 RPM	270°, 7 rms
Case 4	6.55	4.4		10 000 RPM	270°, 7 rms
Case 5	7	4.5	Soft tissue	10 000 RPM	270°, 7 rms
Case 6	6.45	4.1		10 000 RPM	270°, 7 rms
Case 7	6.5	4.8	Deep punch	10 000 RPM	270°, 7 rms
Case 8	6.35	4.1		10 000 RPM	270°, 7 rms
Case 9	6.1	4.5	Deep punch	10 000 RPM	270°, 7 rms
Case 10	5.5	3.7		10 000 RPM	270°, 7 rms
Case 11	6.5	4.4		10 000 RPM	270°, 11 rms
Case 12	7.05	4.5		10 000 RPM	270°, 11 rms
Case 13	6.5	4.3		10 000 RPM	270°, 11 rms
Case 14	6.6	4.3		10 000 RPM	270°, 11 rms
Case 15	6.45	4.5	Soft tissue	10 000 RPM	270°, 11 rms

## 3 RESULTS

Based on the combined results from 15 cases, the average number of hair follicles per graft was 2.029 for the rotary method and 2.084 for the oscillatory method. Although this shows a slight increase for the oscillatory method, the difference was not statistically significant. However, the total yield rate was 88.3% for the rotary group and 90.5% for the oscillatory group (Table 2), with the difference being statistically significant. The *p*‐value for the comparison of average hairs per graft between the rotary and oscillatory methods is 0.0476, and the *p*‐value for the yield rate comparison is 0.0284. Both *p*‐values are below the conventional significance level of 0.05, indicating significant differences in both the average hairs per graft and yield rates between the two methods.

**TABLE 2:** The total yield rate was 88.3% for the rotary group and 90.5% for the oscillatory group, with the difference in means being statistically significant.

Case	Rotary (average hairs/graft)	Oscillatory (average hairs/graft)	Rotary (yield rate)	Oscillatory (yield rate)
Case 1	2.16	2.09	84.00%	89.40%
Case 2	1.95	2.19	84.80%	94.80%
Case 3	2.16	2.11	92.80%	91.60%
Case 4	2.02	2.06	91.50%	91.00%
Case 5	1.71	2.01	83.70%	88.90%
Case 6	1.97	1.96	90.50%	94.50%
Case 7	1.66	1.82	84.40%	90.10%
Case 8	2.08	2.26	93.10%	93.70%
Case 9	2.05	2.22	90.40%	91.00%
Case 10	2.27	2.17	91.50%	91.60%
Case 11	2.14	2.10	90.50%	90.20%
Case 12	2.18	2.22	89.30%	90.80%
Case 13	2.16	2.19	89.50%	88.00%
Case 14	1.99	1.95	89.30%	87.50%
Case 15	1.81	2.15	86.50%	91.50%
Mean	2.029	2.084	88.83%	90.52%
SD	0.186144	0.123624	0.032381	0.02406

Despite these findings, prior research and the current study indicate that the oscillatory method generally yields better harvesting results, especially in cases where the scalp is very soft or when a deeper punch is required than the follicle length. To further investigate this, we selected five cases exhibiting these conditions to form a “Selected Group” and reanalyzed the data, highlighting the advantages of the oscillatory method under these specific circumstances.

In the Selected group, the average number of hairs per graft was 1.836 for rotary and 2.078 for oscillatory, with the difference being statistically significant. The total yield rate was 86% for rotary and 91% for oscillatory, with the difference between the rotary and oscillatory groups also being statistically significant (Table 3). The *p*‐value for the comparison of average hairs per graft between the rotary and oscillatory methods is 0.00236, indicating a statistically significant difference. The *p*‐value for the yield rate comparison is 0.05556, which is not statistically significant at the conventional 0.05 level.

**TABLE 3:** These include cases where the scalp is exceptionally soft or where the punch depth needs to be greater than the follicle length. We selected five such cases to form a “Selected Group” and re‐evaluated the statistics.

Case	Rotary (average hairs/graft)	Oscillatory (average hairs/graft)	Rotary (yield rate)	Oscillatory (yield rate)
Case 2	1.95	2.19	84.80%	94.80%
Case 5	1.71	2.01	83.70%	88.90%
Case 7	1.66	1.82	84.60%	87.80%
Case 9	2.05	2.22	90.40%	91.80%
Case 15	1.81	2.15	90.00%	91.50%
Mean	1.836	2.078	86.00%	90.96%
SD	0.171	0.175	0.00322	0.027373

The yield percentage of the selected group of cases, where the scalp was very soft or the depth of the punch had to be deeper than the length of the hair follicle, was compared, revealing a difference (Table 4). The oscillatory method provided better results in these cases. The transection rate in rotary and oscillatory methods is presented in Table 5.

**TABLE 4:** Yield percentage of selected group of cases, where the scalp was very soft or the depth of the punch had to be deeper than the length of the hair follicle, and then compared the numbers, there was a difference. Oscillatory method provided better results in these cases.

Yield percentage			
		Rotary	Oscillatory
Case 2	Single	23.59%	11.57%
	D.single	10.11%	7.20%
	Double	47.19%	50.13%
	Triple	19.11%	31.10%
Case 5	Single	38.44%	21.22%
	D.single	9.87%	7.08%
	Double	42.08%	49.03%
	Triple	9.61%	22.67%
Case 7	Single	38.51%	24.04%
	D.single	16.55%	15.06%
	Double	40.38%	54.32%
	Triple	4.56%	6.58%
Case 9	Single	16.60%	6.78%
	D.single	11.89%	9.20%
	Double	50%	55.20%
	Triple	21.51%	28.82%
Case 15	Single	33.95%	9.83%
	D.single	9.70%	6.56%
	Double	41.04%	59.01%
	Triple	15.31%	24.60%

**TABLE 5:** Transection rate in rotary and oscillatory methods (transection rates = 100 − total yield rate).

Transection rate		
	Rotary	Oscillatory
Case 1	16.00%	10.60%
Case 2	15.20%	5.20%
Case 3	7.20%	10.00%
Case 4	8.50%	8.10%
Case 5	16.30%	11.10%
Case 6	9.50%	5.50%
Case 7	15.40%	12.20%
Case 8	6.90%	7.00%
Case 9	9.60%	8.20%
Case 10	8.70%	11.40%
Case 11	9.50%	9.80%
Case 12	10.70%	9.20%
Case 13	9.80%	12.90%
Case 14	10.70%	12.50%
Case 15	13.50%	8.50%

## 4 DISCUSSION

The findings of this study demonstrate that while the overall differences between the rotary and oscillatory punch methods in terms of total yield rate and the average number of hairs per graft are minimal, specific conditions such as soft scalp or deeper punch requirements highlight the superiority of the oscillatory method. This distinction is crucial for improving surgical outcomes and tailoring approaches to individual patient needs.

The statistically significant higher total yield rate and average number of hairs per graft with the oscillatory method in selected cases align with existing literature suggesting the advantages of this technique in minimizing follicle damage. The reduced torsional force generated by the small arc of rotation in the oscillatory punch helps preserve the integrity of splayed and curved follicles, which are more susceptible to damage during extraction with continuous rotary motion.^8^, ^9^, ^10^

Adopting a structured guideline before FUE, as adhered to in this study, plays a pivotal role in optimizing outcomes (Table 4). By evaluating hair characteristics and adjusting punch diameter and depth accordingly, the transection rate can be minimized, and the number of hairs per graft can be maximized. This approach underscores the importance of personalized treatment plans in hair transplantation surgery.

The study of Civas et al.^11^ analyzed data from 1415 male patients who underwent FUE hair transplantation at a dermatology clinic between 2011 and 2020, revealing that advancements in punch technology, such as serrated and trumpet punches, have significantly increased the number of total grafts and the proportion of grafts containing three hair follicular units (3FU). As a result, these technological improvements have led to a higher hair yield and made the FUE method the preferred choice for hair transplantation among both doctors and patients, compared to earlier perceptions of its limited applicability.

The observed differences between the rotary and oscillatory methods emphasize the need for surgeons to be proficient in multiple techniques and to select the most appropriate method based on individual patient characteristics. While the rotary method remains effective for many patients, the oscillatory method offers significant benefits in specific scenarios, particularly for patients with soft scalps or those requiring deeper punches (Table 6).

**TABLE 6:** Guideline for pre‐follicle extraction aims to decrease the transection rate and increase the number of transplanted hairs.

Step	Description
Patient's hair characteristics evaluation	Evaluating the characteristics of the patient's hair
Selection of punch diameter size	Choosing the appropriate punch size
Assessment of hair length	Measuring the length of the hair
Adjustment of punch depth	Adjusting the depth of the punch
Adjustment of rotation method	Modifying the rotation technique

Further research with larger sample sizes and diverse patient populations is necessary to validate these findings and refine the guidelines for punch selection in FUE surgery. Additionally, advancements in punch technology and technique refinement could further enhance graft survival rates and overall surgical success.

In conclusion, this study highlights the nuanced differences between the rotary and oscillatory punch methods in FUE surgery. While both methods are generally effective, the oscillatory method demonstrates significant advantages in specific cases, supporting its use as a preferred technique under certain conditions. Implementing structured guidelines and personalized approaches in FUE can lead to improved outcomes, higher graft survival rates, and better patient satisfaction.

## AUTHOR CONTRIBUTIONS

**Jino Kim, Kyu‐Ho Yi, and Yong‐Uk Ko**: Conceptualization. **Jino Kim, Kyu‐Ho Yi**: writing—original draft preparation. **Jino Kim, Kyu‐Ho Yi**: writing—review and editing. Jino **Kim, Kyu‐Ho Yi**: visualization. **Kyu‐Ho Yi**: supervision. All authors have reviewed and approved the article for submission.

## FUNDING INFORMATION

There is no funding supported.

## CONFLICT OF INTEREST STATEMENT

I acknowledge that I have considered the conflict of interest statement included in the “Author Guidelines.” I hereby certify that, to the best of my knowledge, that no aspect of my current personal or professional situation might reasonably be expected to significantly affect my views on the subject I am presenting.

## Data Availability

The data that support the findings of this study are available from the corresponding author upon reasonable request.
